# Non‐exploitative human disturbance provides shelter for prey from predator

**DOI:** 10.1002/ece3.10200

**Published:** 2023-06-15

**Authors:** Saneer Lamichhane, Babu Ram Lamichhane, Aasish Gurung, Trishna Rayamajhi, Tulasi Prasad Dahal, Pramod Raj Regmi, Chiranjibi Prasad Pokheral, Abhinaya Pathak, Ganesh Panta, Ram Chandra Kandel, Madan K. Oli

**Affiliations:** ^1^ National Trust for Nature Conservation Kathmandu Nepal; ^2^ Department of Wildlife Ecology and Conservation School of Natural Resources and Environment University of Florida Gainesville Florida USA; ^3^ Department of Natural Resources and the Environment Cornell University Ithaca New York USA; ^4^ Ministry of Forests and Environment Kathmandu Nepal; ^5^ Department of Wildlife Ecology and Conservation University of Florida Gainesville Florida USA; ^6^ School of Biological Sciences University of Aberdeen Aberdeen UK

**Keywords:** human disturbance, human shield hypothesis, human–prey–predator relationship, multispecies occupancy model, spatial refugia, spatiotemporal overlap

## Abstract

Human activities can influence behaviors of predators and prey, as well as predator–prey interactions. Using camera trap data, we investigated whether or to what extent human activities influenced behaviors of predators (tigers and leopards) and prey (sambar deer, spotted deer, wild boar, and barking deer), and predator–prey interactions in the Barandabhar Corridor Forest (BCF), Chitwan District, Nepal. A multispecies occupancy model revealed that the presence of humans altered the conditional occupancy of both prey and predator species. Specifically, the conditional occupancy probability of prey was substantially higher (*ψ* = 0.91, CI = 0.89–0.92) when humans were present than when humans were absent (*ψ* = 0.68, CI = 0.54–0.79). The diel activity pattern of most prey species overlapped strongly with humans, whereas predators were generally more active when humans were absent. Finally, the spatiotemporal overlap analysis revealed that human–prey interactions (i.e., the probability that both humans and prey species being present on the same grid at the same hourly period) was ~3 times higher (10.5%, CI = 10.4%–10.6%) compared to spatiotemporal overlap between humans and predators (3.1%, CI = 3.0%–3.2%). Our findings are consistent with the human shield hypothesis and suggest that ungulate prey species may reduce predation risk by using areas with high human activities.

## Graphical abstract

The conditional occupancy probability of prey was substantially higher (*ψ* = 0.91, CI = 0.89–0.92) when humans were present than when humans were absent (*ψ* = 0.68, CI = 0.54–0.79). The diel activity pattern of most prey species overlapped strongly with humans, whereas predators were generally more active when humans were absent. Finally, the spatio‐temporal overlap analysis revealed that human‐prey interactions was ~3 times higher (10.5%, CI = 10.4–10.6%) compared to spatio‐temporal overlap between humans‐predators (3.1%, CI = 3.0–3.2%).

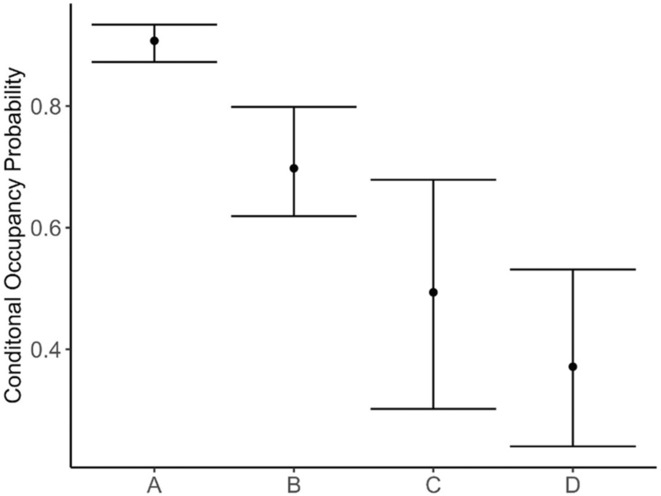

## 1 INTRODUCTION

As the human–wildlife interface continues to shrink due to an ever‐decreasing amount of land unaffected by human disturbance, the frequency and intensity of human–wildlife interactions continue to increase globally (Sih et al., 2011; Thirgood & Redpath, 2008). Human presence and activities such as firewood and fodder collection and traveling along the forest roads and trails can affect the behaviors of predators and prey, and can potentially displace predators, prey, or both (Frid & Dill, 2002; Muhly et al., 2011; Preisser & Bolnick, 2008; Schmitz et al., 1997; Shannon et al., 2014). These human‐induced changes in the behaviors and spatial distribution of predators and/or prey can affect predator–prey interactions, including both the consumptive and non‐consumptive effect of predators on prey (Preisser et al., 2005; Preisser & Bolnick, 2008; Wirsing et al., 2021).

Prey species actively employ various behavioral and morphological defenses to evade predation, demonstrating a repertoire of adaptive responses (Lima & Dill, 1990; Preisser et al., 2005; Preisser & Bolnick, 2008; Sih, 2005). The success of these defensive strategies is contingent upon factors such as available food resources and habitat conditions, which directly impact the survival prospects of prey species (Heithaus et al., 2009). The presence of predators plays a crucial role in shaping the dynamics of prey populations and the intricate interactions within predator–prey systems (Preisser et al., 2005; Preisser & Bolnick, 2008). A well‐documented phenomenon in prey behavior is the risk effect, characterized by a trade‐off where prey reduces their foraging time to minimize vulnerability to predation (Lima & Dill, 1990; Schmitz et al., 1997; Sih, 1984; Skelly & Werner, 1990; Werner, 1991). This trade‐off can result in two potential outcomes: an increased risk of starvation and mortality (lethal indirect effect) or alterations in changes in behavior including habitat selection (nonlethal indirect effect). To mitigate these risks, prey species may strategically utilize vacant domains, selectively choosing specific habitats (e.g., choosing rugged terrain compared to predators) or times of the day when predators are less active or pose a lower level of threat (Kohl et al., 2019). However, it is possible that prey species may deliberately choose habitats near human presence as a means of finding protection from predators. This preference arises from the fact that large predators are typically more sensitive to human presence, making areas with human activity a potential refuge for prey (Muhly et al., 2011; Treves & Karanth, 2003; Woodroffe, 2000).

Existing evidence suggests that prey species tend to have a higher tolerance for human disturbance than predators (Hebblewhite et al., 2005; Mori et al., 2020). For example, Muhly et al. (2011) showed that all large mammalian prey species in the Rocky Mountains of Canada were closer to humans than predators such as wolves (*Canis lupus*) and cougars (*Puma concolor*). This suggests that increased human activities can potentially protect prey from predators (Berger, 2007). In other words, human‐induced disturbance stimuli can create spatial refuges for prey to minimize the risk of predation (Muhly et al., 2011). This idea, dubbed the “human‐shield hypothesis”, posits that herbivorous mammals use humans to shield against predators, which can create a zone with reduced natural predation risk in areas with a high degree of human disturbance (Berger, 2007). For example, elk (*Cervus elaphus*) in Banff National Park, Canada are attracted to areas with high human activity, which is avoided by their main predator, the wolves (Hebblewhite et al., 2005). However, the human shield hypothesis has not been thoroughly tested in human‐dominated landscapes.

**FIGURE 1:**
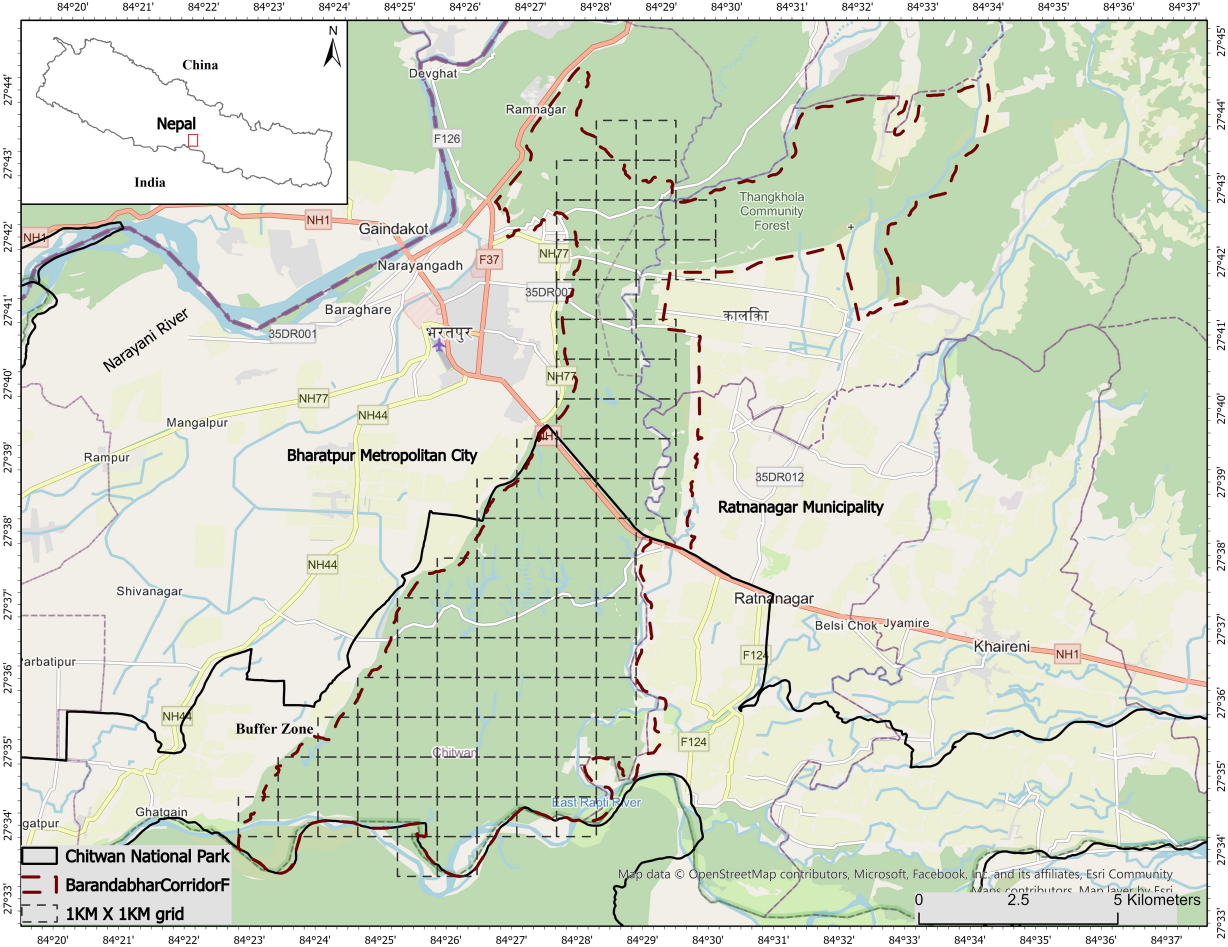
Location of the study area, Barandabhar Corridor Forest, Nepal. Each square represents 1 km × 1 km grid cell where a pair of cameras was placed. The buffer zone is a government‐declared area that provides forest materials to local people.

Our goal was to test the human shield hypothesis in the Barandabhar Forest Corridor (BFC; Figure 1), Chitwan District, Nepal. The BFC is an ecologically important corridor connecting the iconic Chitwan National Park with the Mahabharat range within the Terai Arc Landscape of Nepal. The corridor is inhabited by large predators such as tigers (*Panthera tigris tigris*), common leopards (*Panthera pardus fusca*), and dholes (*Cuon alpinus*), and several ungulate prey species including the sambar deer (*Rusa unicolor*), spotted deer (*Axis axis*), wild boar (*Sus scrofa*), and barking deer (*Muntiacus vaginalis*) (Aryal et al., 2012; Lamichhane et al., 2016). We predicted that (i) the presence of humans will influence the occupancy probability of both prey and predators, with prey being more, and predators less, likely to be present in areas with high human activities, and (ii) prey will have a greater temporal and spatiotemporal overlap with humans compared to that with a predator or that between predators and humans. We expect that our findings regarding the influence of human presence on predator–prey interactions would be helpful in devising strategies for managing human pressure in the forests, minimizing human–wildlife conflict, and promoting coexistence in areas where humans and large mammals cohabit.

## 2 MATERIALS AND METHODS

### 2.1 Study area

The Barandabhar Corridor Forest (BCF) is an 87.9 sq km biological corridor in Nepal connecting the Chitwan National Park, a UNESCO heritage site, to the Mahabharat range to the north (Figure 1). The climate is subtropical with winter, spring, and monsoon seasons, and the vegetation is dominated by Sal (*Shorea robusta*) forest, and by riverine and mixed hardwood forest (Bhattarai, 2003). The BCF harbors at least 33 species of mammals, 372 species of birds, 37 species of fish, and 31 species of herpetofauna (Lamichhane et al., 2016, 2021).

The BCF is bisected by the East–West Highway (Mahendra Highway), one of the busiest national routes in the country, with the southern part located within the buffer zone of the Chitwan National Park; the northern part of BCF is protected under the administration of the Division Forest Office, Chitwan District, Nepal. The BCF is situated between the densely populated Ratnanagar and Kalika municipalities to the east and the Bharatpur Metropolitan City to the west, and experiences high levels of human disturbance, especially in the northern part (Lamichhane et al., 2018).

### 2.2 Camera trap surveys

Camera trap surveys were conducted between December 2015 and January 2016. Using ArcGIS 10.2, grid cells of 1 × 1 km (*n* = 88 grid cells) were overlaid over the study area (Figure 1). At each grid, a pair of unbaited camera traps were placed along the forest roads and trails at strategic locations based on the presence of footprints, scats, or urine of both prey and predator species. We used the Reconyx 550 (Reconyx Inc, Holmen, Wisconsin, USA), Moultrie 40D (Moultrie Products LLC, Birmingham, Alabama, USA), Bushnell HD (Bushnell Outdoor Products, Overland Park, Kansas, USA), and Cuddeback (Cuddeback Digital, Greenbay, Wisconsin, USA) digital motion sensor camera traps. In each grid, camera traps were installed at 0.5 m above ground, 4–5 m apart on both sides of the trails (Claridge et al., 2004; Rovero et al., 2013). The GPS coordinates were recorded for each camera, and each camera was configured to record the date and time in the image shot. The camera traps were set to the most sensitive setting with a short delay between image captures (FAP – Fast as Possible), and they remained in place concurrently for a minimum of 20 days, totaling 1760 trap nights.

### 2.3 Data analysis

We considered four species of ungulate prey: sambar deer, spotted deer, barking deer, and wild boars; and two species of predator species: tigers and leopards, and humans. We calculated the naïve occupancy of each species by dividing the number of species detected grids by the total number of grids (Mackenzie et al., 2017).

#### 2.3.1 Multispecies occupancy model

We used the multispecies occupancy model (MSOM) to examine segregation (or conversely, overlap) in space use between predators and prey, both in the presence and absence of humans (Rota et al., 2016). We grouped camera trap species into prey (sambar deer, spotted deer, wild boar, and barking deer) and predator (tiger and leopard) guilds. This allowed us to analyze information across the guilds while reducing the number of parameters to be estimated (Van der Weyde et al., 2018). Using camera trap data, we created a detection history matrix with each row corresponding to a site or a grid cell (*n* = 88 rows), and each column corresponding to 1 day of survey (*n* = 20 columns). If a member of the prey (or predator) guild was detected in a camera trap in a particular site on a particular survey occasion, it was coded ‘1’; it was coded ‘0’ otherwise. For example, if a species (or guild) was camera‐trapped every other day during a survey consisting of five consecutive days (occasions), then its detection history would be 10101 (MacKenzie et al., 2002).

The MSOM estimates the probability of detecting a species (*p*) given that it is present at a site, and the probability (*ψ*) of various combinations of the presence and absence of two or more interacting species. The probability of different combinations of presence and absence of ‘*S*’ focal species is modeled using a multinomial logit link and natural parameters. When *S* = 2, first‐order natural parameters (*f*_1_ and *f*_2_) represent the probability of occurrence of species 1 and 2, respectively, conditional on the absence of the other species; and a second‐order natural parameter (*f*_12_) represents the probability of occurrence of the two species together (Rota et al., 2016).

We used the R package ‘unmarked’ to fit two a priori MSOM models: a model with no species interaction (first‐order natural parameter), and a second model that allowed pairwise species interactions (i.e., prey or predator occupancy in the presence and absence of humans (second‐order natural parameters) (Fiske & Chandler, 2011, 2019; Rota et al., 2016)). We compared the two models based on the Akaike Information Criterion (AIC) and chose the model with the lower AIC value for parameter estimation (Burnham & Anderson, 2002). Then, we calculated the marginal probability for the occurrence of guild *S*, the probability of occurrence of human–prey and human–predator guilds, and the probability of occurrence of prey or predator guilds, conditional on the presence or absence of humans (Rota et al., 2016).

#### 2.3.2 Temporal overlap analysis

We applied the temporal overlap analysis to test for the overlap in diel activity pattern between the humans and prey, and humans and predators. For these analyses, we considered each prey or predator species separately (as opposed to prey or predator guild) because different species of prey (or predator) exhibited different diel activity patterns. We avoided the inflated counts by considering a single detection of a species within 15 min as an independent capture event for the entire survey (Keim et al., 2019). Then, using the ‘overlap’ package in R, we estimated the pairwise coefficient of overlap (Δ) (human–prey species and human–predator species) to examine the overlap in activity pattern (Ridout & Linkie, 2009). The coefficient of overlap determines the amount of overlap between two kernel densities by utilizing the minimum density function from two sets of data that are being compared at various times. The area under both density curves, ranging from 0 (no overlap) to 1 (complete overlap), was used to define an overlap (Linkie & Ridout, 2011). The overlap was interpreted as Δ < 0.5 as “low overlap”, 0.5 > Δ < 0.75 as “moderate overlap”, and > 0.75 as “high overlap”. We chose the Δ_4_ and Δ_5_ estimators because it was recommended for sample sizes >75 observations and between >50 and <75 observations (leopard in our case), respectively. Additionally, we used the bootstrap method with 1000 resamples to obtain the 95% confidence interval for each overlap index (Meredith et al., 2014).

#### 2.3.3 Spatiotemporal overlap

We used the spatiotemporal overlap method proposed by Karanth et al. (2017) to infer overlap in both space and time simultaneously. For each grid, we created a matrix of species detection per hour, with rows representing the grid and columns representing hourly intervals of the diel cycle. Each cell of the matrix contained the total number of detections of a predator or prey guild at a given grid during a specific hourly interval, aggregated over the entire survey (Karanth et al., 2017; Zanni et al., 2021). We then calculated the proportion of grids where: (i) each species was detected alone in the absence of any other species, (ii) the presence of two species overlapped, and (iii) all three species were active at each hourly interval. Using the bootstrap method with 1000 resamples, we estimated the standard error and 95% confidence intervals for the spatiotemporal overlap of one guild with another and with humans. We also plotted Venn diagrams using the ‘Euler’ package in R (Larsson, 2022) to visualize the spatiotemporal overlap of human, prey, and predator guilds.

## 3 RESULTS

During a 20‐day camera trap survey, we recorded a total of 2050 independent capture photos of prey, predator, and human. The most frequently captured species were humans (*n* = 1065), followed by spotted deer (*n* = 475), wild boar (*n* = 127), barking deer (*n* = 127), sambar deer (*n* = 124), tigers (*n* = 80), and leopards (*n* = 52). The naïve occupancies were human: 0.36, sambar deer: 0.47, spotted deer: 0.67, wild boar: 0.42, barking deer: 0.36, tiger: 0.35, and leopard: 0.26.

### 3.1 Spatial overlap

**FIGURE 2:**
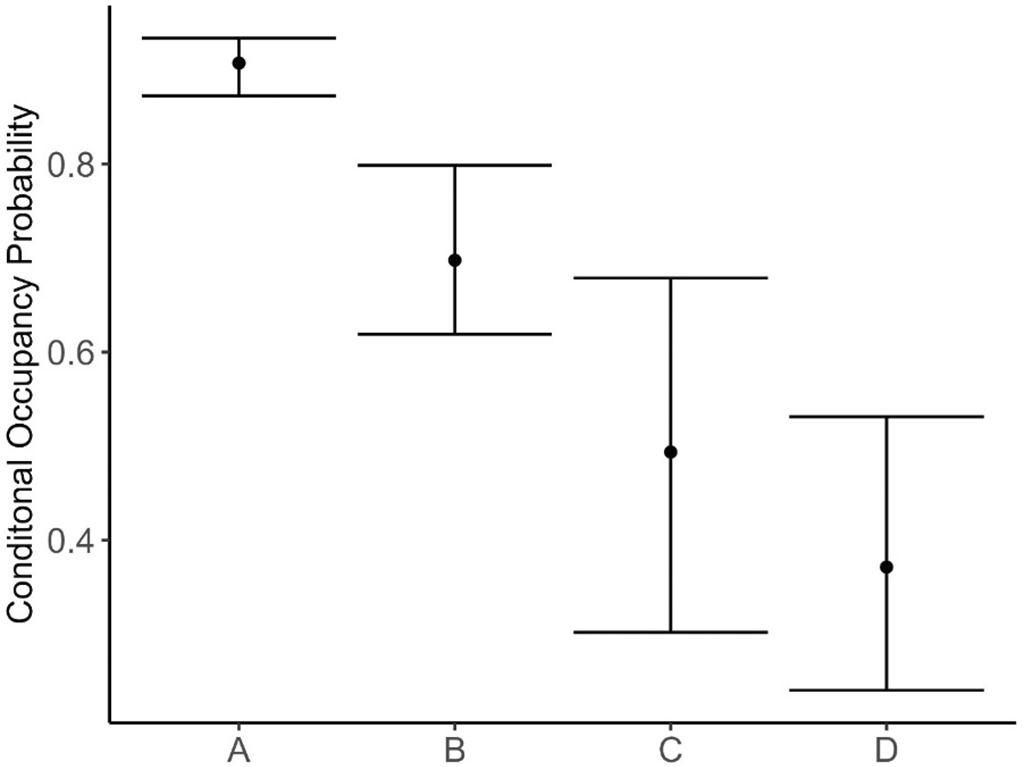
Conditional occupancy probability estimates with 95% confidence intervals for prey and predator guilds given the presence or absence of humans. Here: A = the conditional occupancy probability of the prey guild given the human presence, B = the conditional occupancy probability of the prey guild given the human absence, C = the conditional occupancy probability of the predator guild given the human presence, and D = the conditional occupancy probability of the predator guild given the human absence.

The MSOM revealed that the paired interaction model (AIC = 3380) was better supported than the no interaction model (AIC = 3389) (Appendix 1). Based on the top model, the marginal occupancy probability of prey and predator guilds, and the humans was 0.78 (CI = 0.71–0.84) and 0.42 (CI = 0.29–0.58), and 0.38 (CI = 0.30–0.46), respectively. Similarly, the probability of occupancy of prey and predator guilds with humans was 0.35 (CI = 0.26–0.47) and 0.19 (CI = 0.13–0.29), respectively. Furthermore, the results indicated that, in the presence of humans, both prey and predator guilds showed changes in their conditional occupancy probability, with a significantly higher occupancy probability of the prey guild when humans were present. However, the 95% confidence range for the conditional occupancy of predator guild when humans were present overlapped with that when humans were absent, indicating a lack of significance in this effect (Figure 2).

### 3.2 Temporal overlap

**FIGURE 3:**
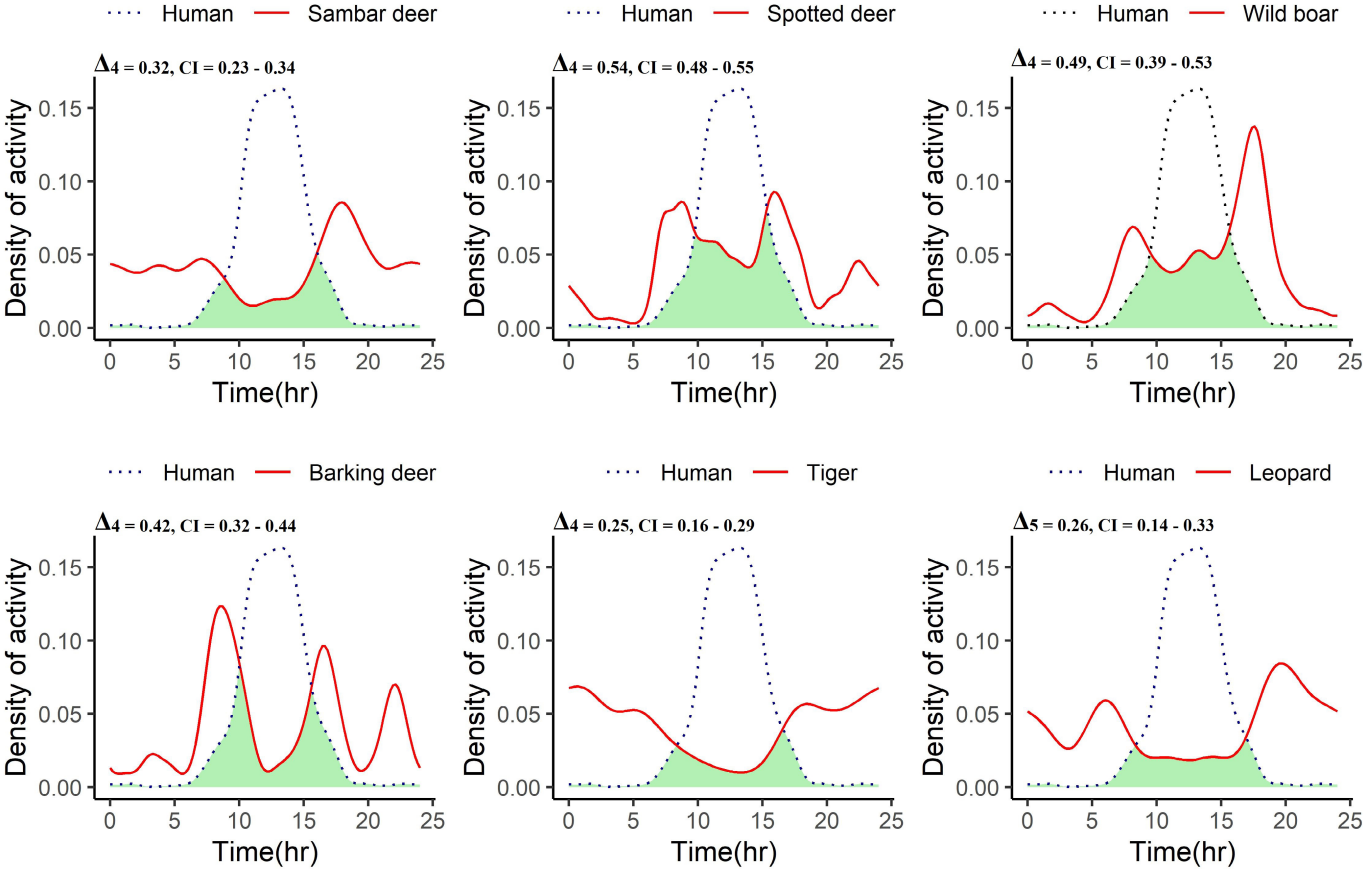
Temporal overlap in diel activity pattern of prey and predator species with humans. The *x*‐axis represents the hourly interval over a 24‐h period, and *y*‐axis represents the kernel density of activity. The values on the top left corner represent the coefficients of overlap and 95% confidence intervals when the sample size was >75 (Δ_4_) and when the sample size was between 50 and 75 (Δ_5_).

Different species exhibited different diel activity patterns. Barking deer and leopards were primarily crepuscular; spotted deer and wild boar were primarily diurnal whereas tigers and sambar deer were primarily nocturnal. The activity pattern of humans strongly overlapped with that of spotted deer (Δ_4_ = 0.54, CI = 0.48–0.55), and wild boar (Δ_4_ = 0.49, CI = 0.39–0.53). There was little overlap in activity patterns between humans and leopards (Δ_5_ = 0.26, CI = 0.14–0.33), and humans and tigers (Δ_4_ = 0.25, CI = 0.16–0.29) (Figure 3).

### 3.3 Spatiotemporal overlap

**FIGURE 4:**
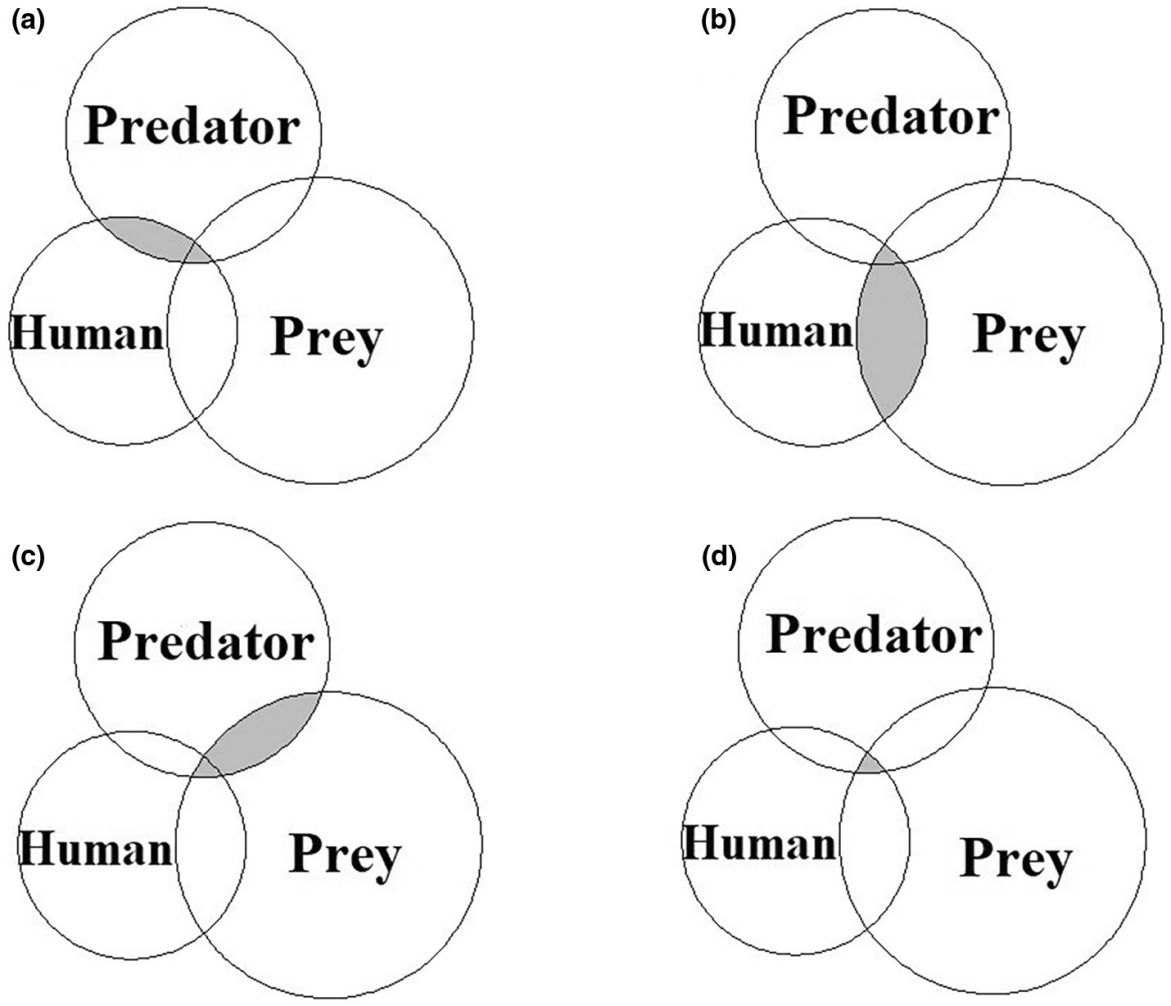
Proportion of space and time in which the activity of human–predator guild (a), human–prey guild (b), and predator–prey guilds (c) overlapped, and the proportion of space–time where the activity of human, prey, and predator overlapped (d).

The proportion of space and time that humans, predator, and prey guilds were exclusively active was 0.380 (CI = 0.370–0.384), 0.558 (CI = 0.554–0.562), and 0.772 (CI = 0.770–0.774), respectively. There was a substantially greater spatiotemporal overlap between humans and prey (0.105, CI = 0.104–0.106) than between humans and predators (0.031, CI = 0.030–0.032) or predators and prey (0.060, CI = 0.059–0.061). Likewise, the overlap between humans, predator, and prey guilds together at the same grid and the same hourly time was 0.0085 (CI = 0.008–0.009) (Figure 4).

## 4 DISCUSSION

Barandabhar Corridor Forest (BCF) is an ecologically important biological corridor because it provides connectivity between the Mahabharat range and the iconic and biodiverse Chitwan National Park, Nepal (Lamichhane et al., 2018). Though the BCF is relatively small (~88 sq km), the corridor is home to diverse species such as tigers and leopards, and their prey species such as sambar deer and spotted deer. But BCF is surrounded by heavily populated municipalities. Local people rely on the biological corridor for firewood and fodder (Lamichhane et al., 2016, 2021). The high level of human activities was evident in our camera trap data because humans were the most frequently photographed species. We sought to quantify whether or to what extent a high degree of human activities in BCF affected the behavior of predator and prey species or predator–prey interactions.

We found that the conditional occupancy of a site by prey was significantly higher when humans were present compared to when humans were absent. We did not differentiate the type of humans such as tourists on safari or local community members in the forest for resource collection. The tourists are interested in seeing wildlife and could purposefully be visiting the areas with a high probability of sighting these ungulates. The southern (Buffer Zone) part of the corridor is a popular destination for jungle walk, increasing the chances of overlapping with prey species. In contrast, predators exhibited little spatial overlap with humans, and human presence did not significantly influence their occupancy probability. Barber‐Meyer et al. (2013) and Karanth et al. (2011) also reported a weaker influence of human disturbance on tiger occupancy in the lowlands of Nepal and India, respectively. We note, however, tigers and leopards can potentially persist in human‐dominated areas with their diet partially supplemented by livestock (Athreya et al., 2015; Karanth & Gopal, 2005; Kumbhojkar et al., 2021), indicating the potential for localized adaptation of these large predators to human presence.

Human presence can also influence predator–prey interaction by altering their diel activity patterns (Petridou et al., 2023). Analysis of activity patterns revealed that prey species showed high temporal overlap with humans, whereas there was little overlap in the activity of predators with that of humans. It suggests that most of the prey species did not avoid each other or humans because they were active at the same time; a high degree of temporal overlap in activity patterns has also been reported by Mori et al. (2020) and Zanni et al. (2021). Furthermore, the temporal overlap between the activity pattern of predators and humans was low, which was consistent with the findings of Blašković et al. (2022), who also found low overlap in the activity patterns of humans and predators. While Lamichhane et al. (2019) found that both predators display crepuscular behavior in the adjacent Chitwan National Park, our study demonstrated that tigers and leopards have distinct nocturnal and crepuscular activity patterns in BCF. Like our findings, Carter et al. (2012) suggested that tigers may have temporally altered their activity pattern to avoid humans in BCF.

Finally, human presence can simultaneously affect predators and prey in space and time. Our results of spatiotemporal analyses revealed that the proportion of the space and time overlap between prey and humans was high compared to prey and predators, indicating that the spatiotemporal overlap may significantly influence the coexistence of these species in the presence of humans. The results indicate that prey may use areas with human presence as antipredator behavior to reduce predation risk (Abbey‐Lee et al., 2016; Laundré et al., 2010; Lima & Dill, 1990; Preisser et al., 2005; Preisser & Bolnick, 2008; Sih, 2005). Prey density in the BCF is high (14,400/100 sq km; Chaudhary, 2020) and the density of the top predator, the tiger, also is high (9/100 sq km; NTNC, 2015); it is likely that tigers adjust their spatial and temporal behavior to increase their chances of encountering prey (Carbone et al., 2007; de Matos Dias et al., 2018; Kuijper et al., 2013; Laundré et al., 2010; Monterroso et al., 2013). Prey being an active player minimizes the risk of predation by opportunistically maximizing spatial or spatiotemporal overlaps with humans compared to their lethal counterparts – predators. The prey trade off this advantage for activities such as feeding and reproduction (Lamichhane & Jha, 2015; Stephens, 1987). Humans do not directly harm the prey species as hunting is restricted in these forests. Therefore, human presence appears to mediate the interaction between prey and predator both in space and time, with prey being more successful in avoiding predators (Sih, 2005).

Taken together, our findings suggest that in areas with hunting restrictions, human presence reduces the risk posed to prey by predators, and provides support for the human shield hypothesis, which proposes that prey prefer spatial refugia created by human disturbance as a shield against predators (Berger, 2007). This reduction in predation risk may create a reduced vigilance in prey species (Valeix et al., 2009) and increased spatial and temporal avoidance of predators (Thaker et al., 2011; Vanak et al., 2013). Other studies reported similar results (Gehr, 2016; Muhly et al., 2011; Nickel et al., 2020; Smith et al., 2018). Additionally, the use of human presence by prey as a shield impacts key factors that shape the landscape of fears, such as considering predator presence when choosing feeding areas and predicting attack likelihood (Berger, 2007; Bleicher, 2017). This interaction of prey with humans to escape predation may lead to trophic cascading and resource depletion (Schmitz & Suttle, 2001). Hence, reducing human presence in the BCF could possibly decrease the trophic effect and increase the area available for predators (Musiani et al., 2010; Rogala et al., 2011). Whereas our results are consistent with predictions arising from the human shield hypothesis, we note that our study suffered from some potential limitations. For example, our study was conducted over a short period of time in a fairly small area, and our analyses were based on limited sample sizes. Thus, our study did not capture seasonal and/or annual variation in predator–prey activity patterns, and their response to human presence. Finally, we note that we cannot conclusively reject alternative explanations of the patterns observed in our study. For example, the observed pattern could arise if prey species preferentially use habitats where humans collect firewood and fodder. Long‐term studies in larger spatial and temporal scales across a range of human activities would be needed to conclusively reject alternative explanations of observed patterns.

## 5 CONCLUSION

Our study in the Barandabhar Corridor Forest (BCF) has demonstrated an example of prey species taking advantage of human presence to protect themselves from predators, while predators avoid humans in spatiotemporal or temporal overlap rather than in actual space. We observed that prey species overlap more frequently with humans than with predators demonstrating the high adaptive capacity of prey to human presence. On the other hand, the predator's low spatiotemporal overlap with humans illustrates that they actively avoid human presence in the same grid and at the same hourly time. This study highlights the non‐consumptive impacts of human presence on predator–prey relationships, potentially leading to a shift in favor of prey in the predator–prey space race. However, more research is needed to understand the various non‐consumptive effects of human activities on predator–prey interactions. Based on our findings, we encourage wildlife managers and policymakers, including the Buffer Zone User Committees and Buffer Zone Community Forest User Groups to consider the indirect impacts of additional tourists and resource extraction activities on prey–predator interactions in the BCF.

## AUTHOR CONTRIBUTIONS

**Saneer Lamichhane:** Conceptualization (lead); data curation (equal); formal analysis (lead); investigation (lead); methodology (lead); software (lead); validation (equal); writing – original draft (lead); writing – review and editing (lead). **Babu Ram Lamichhane:** Conceptualization (equal); data curation (equal); formal analysis (equal); funding acquisition (lead); investigation (equal); methodology (equal); resources (equal); writing – original draft (equal). **Aashish Gurung:** Conceptualization (equal); data curation (equal); methodology (equal); resources (equal); writing – original draft (equal). **Trishna Rayamajhi:** Conceptualization (equal); formal analysis (equal); methodology (equal); validation (equal); writing – original draft (equal). **Tulasi Prasad Dahal:** Data curation (equal); formal analysis (equal); investigation (equal); resources (equal); writing – original draft (equal). **Pramod Raj Regmi:** Data curation (equal); formal analysis (equal); investigation (equal); resources (equal); visualization (equal); writing – original draft (equal). **Chiranjibi Prasad Pokheral:** Conceptualization (equal); data curation (equal); formal analysis (equal); funding acquisition (lead); methodology (equal); project administration (lead); software (equal); supervision (equal); writing – original draft (equal). **Abhinaya Pathak:** Conceptualization (equal); investigation (equal); methodology (equal); resources (equal); writing – original draft (equal). **Ganesh Panta:** Conceptualization (equal); formal analysis (equal); investigation (equal); resources (equal); supervision (equal); validation (equal). **Ram Chandra Kandel:** Conceptualization (equal); data curation (equal); investigation (equal); methodology (equal); project administration (lead); resources (equal); supervision (equal); writing – original draft (equal). **Madan K. Oli:** Conceptualization (equal); data curation (equal); formal analysis (equal); methodology (equal); software (equal); supervision (lead); validation (lead); writing – original draft (equal); writing – review and editing (equal).

## FUNDING INFORMATION

This study was funded by US Fish and Wildlife Services – Rhino Tiger Conservation Fund (Grant F13AP00825).

## CONFLICT OF INTEREST STATEMENT

There are no competing interests declared by any of the authors.

## Data Availability

The data concerning the endangered tigers and leopards can be obtained upon request, while the remaining data are accessible through the Dryad repository: https://doi.org/10.5061/dryad.kkwh70s8v.

## APPENDIX 1

The estimates of: (A) occupancy and (B) detection probability parameters based on multispecies occupancy models with species interactions. Also presented are the standard error (SE) and *p*‐value testing the null hypothesis that the value of the parameter does not differ from zero. ‘:’ symbol indicates interaction effect.

**Table:**

	Estimate	SE	*p*(>|z|)
**(A)**			
Intercept – human	−1.745	0.14	1.16E−35
Intercept – predator	−1.195	0.252	0.00000208
Intercept – prey	0.536	0.279	0.0547
Human: predator	0.33	0.361	0.361
Human: prey	1.377	0.221	4.35E−10
Predator: prey	0.92	0.307	0.00273
**(B)**			
Human	−0.0186	0.188	0.921
Predator	−2.2131	0.219	6.17E−24
Prey	−0.7305	0.122	2.08E−09
