# CORRIGENDUM

**DOI:** 10.1002/ece3.10247

**Published:** 2023-07-04

In the recent article by Lamichhane et al. (2023), the authors would like to update the Acknowledgments section to read as follows:
